# P-450. Burden of Fungal Infection among People Living with HIV (PLHIV): A Nationwide Inpatient Data Analysis (2019-2021)

**DOI:** 10.1093/ofid/ofae631.650

**Published:** 2025-01-29

**Authors:** Chef Stan L Macaraeg, Kim Abbegail Aldecoa

**Affiliations:** University of Connecticut School of Medicine, Hartford, Connecticut; Trinity Health Oakland/Wayne State University, Pontiac, Michigan

## Abstract

**Background:**

Fungal diseases represent a significant healthcare concern in the general population. Their prevalence and impact are even more pronounced among PLHIV, yet data within this population remains limited. The study aims to investigate the most prevalent fungal infection among PLHIV and its corresponding healthcare burden. Additionally, it explores the sociodemographic of fungal infection among PLHIV.

**Methods:**

The National Inpatient Sample database from 2019 to 2021 was utilized to identify all patients with a primary diagnosis of fungal infection. Patients were categorized into HIV and non-HIV. We used Stata 18.0, with a p-value < 0.05 indicating statistical significance.

**Results:**

Among the 66, 225 hospitalized patients with fungal infection, there were 3543 weighted HIV patients (4.9%). HIV patients were significantly younger (43.8 vs. 62.9), Black (52.7%), with Medicaid (51.2%), and with a low median income of ≤ $49,000 (50.2%). No specific trend was observed from 2019 to 2021 in the prevalence of fungal infections among PLHIV (4.9%, 5.1%, 4.7%) and total hospital costs ($25600, $26913, $25869)

There remains an increased likelihood of cryptococcus (OR: 5.69, 95% CI: 4.6-7.0) and pneumocystis (OR: 10.71, 95% CI: 8.7-13.2) among PLHIV compared to the general population after adjusting for age and sex. Cryptococcus infection has a longer length of stay (LOS) compared to other fungal infections among PLHIV (13.5 vs. 7.6, p< 0.05) and higher total hospital costs ($52470 vs. $20153, p< 0.05). The result was consistent with multivariate analysis after adjusting for age, sex, and Charlson comorbidity index (CCI).

In contrast, pneumocystis infection among PLHIV had shorter LOS compared to other fungal infections (8.3 vs. 9.8, p< 0.05), and the total hospital costs were significantly lower ($21331 vs. $33542, p< 0.05). However, in a multivariate analysis after adjusting for age, sex, and CCI, only the difference in total hospital costs remained significant.

**Conclusion:**

Fungal diseases remain a serious concern among PLHIV, presenting with unique sociodemographic characteristics and significant healthcare costs, particularly with cryptococcus infection. Future strategies should be directed at ways to reduce this significant healthcare burden.

**Disclosures:**

**All Authors**: No reported disclosures

